# Assessment and management of the communication difficulties of children with cerebral palsy: a UK survey of SLT practice

**DOI:** 10.1111/1460-6984.12138

**Published:** 2015-02-04

**Authors:** Rose Mary Watson, Lindsay Pennington

**Affiliations:** Institute of Health and Society, Newcastle UniversityNewcastle upon Tyne, UK

**Keywords:** Cerebral palsy, survey, communication, children, assessment, intervention

## Abstract

**Background:**

Communication difficulties are common in cerebral palsy (CP) and are frequently associated with motor, intellectual and sensory impairments. Speech and language therapy research comprises single-case experimental design and small group studies, limiting evidence-based intervention and possibly exacerbating variation in practice.

**Aims:**

To describe the assessment and intervention practices of speech–language therapist (SLTs) in the UK in their management of communication difficulties associated with CP in childhood.

**Methods & Procedures:**

An online survey of the assessments and interventions employed by UK SLTs working with children and young people with CP was conducted. The survey was publicized via NHS trusts, the Royal College of Speech and Language Therapists (RCSLT) and private practice associations using a variety of social media. The survey was open from 5 December 2011 to 30 January 2012.

**Outcomes & Results:**

Two hundred and sixty-five UK SLTs who worked with children and young people with CP in England (*n* = 199), Wales (*n* = 13), Scotland (*n* = 36) and Northern Ireland (*n* = 17) completed the survey. SLTs reported using a wide variety of published, standardized tests, but most commonly reported assessing oromotor function, speech, receptive and expressive language, and communication skills by observation or using assessment schedules they had developed themselves. The most highly prioritized areas for intervention were: dysphagia, alternative and augmentative (AAC)/interaction and receptive language. SLTs reported using a wide variety of techniques to address difficulties in speech, language and communication. Some interventions used have no supporting evidence. Many SLTs felt unable to estimate the hours of therapy per year children and young people with CP and communication disorders received from their service.

**Conclusions & Implications:**

The assessment and management of communication difficulties associated with CP in childhood varies widely in the UK. Lack of standard assessment practices prevents comparisons across time or services. The adoption of a standard set of agreed clinical measures would enable benchmarking of service provision, permit the development of large-scale research studies using routine clinical data and facilitate the identification of potential participants for research studies in the UK. Some interventions provided lack evidence. Recent systematic reviews could guide intervention, but robust evidence is needed in most areas addressed in clinical practice.

**What this paper adds?**

Children with CP have wide-ranging speech and language impairments and communication needs UK guidelines exist on the areas of need that should be assessed and managed by SLTs, but lack detail on how this should be done. UK SLTs assess and intervene in the areas recommended by clinical guidelines. The most frequently used assessment methods are observation and schedules developed by the therapists themselves, which prevent comparisons across services. Some interventions employed have no supporting evidence base.

## Introduction

‘Cerebral palsy’ (CP) is an umbrella term referring to non-progressive motor disorders that arise from damage to the foetal or infant brain. Children with CP often have limitations in cognition, sensation, communication, and eating and drinking (Rosenbaum *et al*. 2007). Young people with CP who have communication difficulties frequently experience more limited participation in social, educational and community life and have lower perceived quality of life than their peers without communication limitations, including those with CP (Fauconnier *et al*. 2009, Dickinson *et al*. 2007), putting them at severe risk of educational failure and later unemployment. Speech and language therapy (SLT) for children and young people with CP aims to promote their development of effective communication and language systems (Taylor-Goh 2005, RCSLT 2006). How UK SLT services are provided to achieve these aims is currently unclear. It is probable that assessment and intervention practices vary as: health and social care resources for children with special educational needs differ across the UK (Bercow 2008); young people with CP may be educated in segregated schools/units or attend mainstream schools across which expertise in speech, language and communication needs will vary; and although new evidence for individual interventions is emerging, much of our evidence is old and we still lack robust, fully powered randomized controlled trials on which to base strong recommendations for clinical practice (Novak *et al*. 2013). This study aims to explore potential variation in communication assessment and intervention practices of SLTs in the UK, in order to inform service development and research priorities. It is acknowledged that SLT also aims to support the acquisition of eating and drinking skills and to ensure adequate nutrition. Indeed, eating and drinking difficulties and communication needs may be managed by the same SLT. This survey also aimed to explore how the two needs are prioritized. However, detailed investigation of assessment and management of eating and drinking difficulties are beyond the scope of this study.

### Speech

Speech disorder in CP is often associated with underlying motor disorders; approximately 35% of young people with CP have dysarthria (Parkes *et al*. 2010). The prevalence of other developmental speech disorders has not been examined in epidemiological studies to date. Dysarthria in CP commonly affects all aspects of speech production: respiration, phonation, resonance, articulation and prosody. Impairments include: shallow, irregular breathing for speech; low pitched, harsh voice; reduced pitch variation/unexpected pitch breaks; hyper-nasality; and poor articulation (Jeng *et al*. 2006, Ansel and Kent 1992, Ciocca *et al*. 2004, Clark 2003). Although the underlying motor disorders vary (spasticity is associated with increased tone and reduced range of movement, choreo-athetosis is associated with involuntary movement and dysrhythmia), most characteristics (low pitch, poor breath control, imprecise articulation) affect the speech of children with both spastic and dyskinetic CP (Workinger and Kent 1991). An effect of the degradation of the speech signal in dysarthria is to reduce the intelligibility of children's speech, which in turn can lead to difficulties in communication. However, the prevalence of different severities of dysarthria and resulting intelligibility limitations is currently unknown (Hidecker *et al*. 2009).

UK clinical guidelines recommend the use of interventions to reduce speech impairment or improve the physiological support for speech in order to increase speech intelligibility and thereby facilitate the social participation of people with dysarthria (RCSLT 2006, Taylor-Goh 2005). However, the evidence underpinning these recommendations involved adults with acquired dysarthria and may not be directly transferable to children, whose speech and language systems are still developing. A more recent systematic review (Pennington *et al*. 2009a) suggested that physiological approaches focussing on respiratory support and speech rate, which follow motor learning principles, show promise for children and young people with CP. Research covered in that review and in a small number of studies published subsequent to its publication have demonstrated positive effects of the interventions on speech intelligibility, speech loudness and communication change in everyday interactions. Pennington *et al*. (2010, 2013) studied two small groups of young people with mild–severe dysarthria aged 5–17 years, who had different types and severities of motor disorders, some of whom also had intellectual disability. Overall, the young people increased their absolute percentage intelligibility by around 10–15% in single-word and connected speech and gains in intelligibility were maintained at follow-up at 6 and 12 weeks post intervention. The young people also engaged in more interaction activities following therapy (Pennington *et al*. 2013). Fox and Boliek (2012) examined the use of Lee Silverman Voice Therapy Loud® (Sapir *et al*. 2006) with small samples of children with spastic type CP and found that that speech was judged as sounding more natural and speech volume increased after treatment. Robust, fully powered trials are now needed to test the interventions’ clinical and cost-effectiveness and their introduction into clinical practice.

Other approaches to reducing speech impairments in paediatric dysarthria have less supporting evidence. PROMPT (Hayden and Square 1994), a sensori-motor therapy in which therapists provide auditory, visual, tactile and kinesthetic cues for the production of speech sounds, has recently been tested with a small number of single cases. Children have shown changes in lip and jaw control and three out of six children reported also demonstrated gains in intelligibility (Ward *et al*. 2013). Articulation therapy is not generally advised as a first line of treatment, due to the impaired control of multiple speech subsystems in CP dysarthria and the need for breath support to create clear vocal signal (RCSLT 2006, Strand 1995). However, electropalatography (EPG) has been used successfully to remediate residual articulatory errors in older children with CP (Nordberg *et al*. 2011, Gibbon and Wood 2003).

To our knowledge, there is no research evidence that oromotor therapy to increase control of non-speech movements has an impact on speech intelligibility (Pennington *et al*. 2009a) and motor learning theory cautions against such an approach (Ruscello 2008, Wilson *et al*. 2008, Powell 2008a). However, surveys of speech and language therapists have found that non-speech oromotor exercises are frequently used in paediatric SLT in North America (Lof and Watson 2008, Hodge *et al*. 2005).

### Language

Around 48% of children with CP have cognitive impairments, which range in severity from mild to profound (Surman *et al*. 2009). Current research suggests that language difficulties in CP are associated with nonverbal cognitive development rather than comprising specific impairments of language processing. However, current evidence is derived from one sample of children with spastic type CP caused by periventricular leukomalacia (Pirila *et al*. 2007) and one epidemiological study of preschool children (Pennington *et al*. 2012). Further population-based studies are needed to assess language outcome in later childhood. Such research should examine receptive and expressive language and semantics as well as syntax, as lack of experience of the world due to mobility restriction may limit vocabulary.

In both research and clinical practice the methods used to assess language must be carefully considered. Children with restricted upper limb control may have problems manipulating toys in early language tests and children with visual impairments may find it difficult to scan and discriminate between small line drawings. A computer-based receptive language test that can accessed using a touch screen, eye tracking or switches has recently been validated and normed in the Netherlands for children aged 1–6 years (Geytenbeek *et al*. 2014) and this tool may help us to overcome problems associated with toy manipulation in the future. However, it has not yet been translated and tested for use in other languages and countries. Children's understanding of later developing language constructs is more easily assessed, as tests for older children typically require children to select pictures which children with CP can point to using their finger, hand or eyes. Expressive language testing can be accomplished using standard procedures if children have intelligible speech, but for those with limited speech intelligibility it can be difficult to distinguish speech from linguistic errors. For children and young people who are nonverbal and who use alternative and augmentative (AAC) systems accurate assessment of expressive language is particularly challenging and to our knowledge no clear approach has been developed to meet this need (Soto 1999).

Adaptation of test responses (e.g. a clinician may point to each picture in a multiple choice picture-based test and the child indicate when the target picture is reached) can allow children with limited movement and speech to access standard test materials. But, adaptations will invalidate test standardization. Furthermore, some adaptations may entail additional cognitive processing and adapted assessments will therefore no longer be testing the same skills as the original (Warschausky *et al*. 2012). Thus, it is important that researchers and clinicians report how they have presented materials to children with CP when they report results from published tests. It should also be borne in mind that the repeated responses demanded in testing may fatigue children with CP and several short assessment sessions may be needed to gain accurate representation of children's skills.

A review of the evidence for language interventions (Taylor-Goh 2005) showed that experimental studies exist to support intervention for vocabulary, grammar, narrative and social use of language. But, as with test materials, interventions may need to be adapted to meet the individual needs of children with restricted speech and movement and the impact of such adaptations on cognitive processing must be considered.

### Communication

Speech and language are used for the purpose of communication, that is, the sending and receiving of messages between at least two people. Face to face communication may also be accomplished using facial expression, gesture and body movements, but these signals too may be difficult for children with CP to produce. Parents who find their children's communication hard to interpret have been observed to shape conversation around signals that they can understand. However, this limits interaction and creates restricted opportunities for children to develop further communication skills. Children with CP, including those who develop speech, have been observed to take a respondent role in interaction and to use communication for a smaller range of functions than children without motor disorders (Clarke *et al*. 2011, Pennington and McConachie 2001; Voorman *et al*. 2010).

Failure to develop a full range of communicative functions (e.g. the ability to ask questions or signal lack of understanding of a speaker's message) can severely limit children's independence. Early SLT intervention therefore often focuses on training parents to recognize children's idiosyncratic communication signals and to facilitate their children's communication development by creating more frequent and more varied conversational opportunities. Parent communication training has a growing body of experimental evidence to support its implementation (Granlund *et al*. 2008, Pennington *et al*. 2009b) but the generalizability of training has not been tested in randomized controlled trials.

The aim of such early intervention is to provide children with the communication skills onto which they can build language and become independent communicators. For children whose speech intelligibility is severely limited by their motor disorder language may be expressed using AAC. Evidence of the effectiveness of AAC in promoting communicative independence is available for children with wide ranging communication profiles (Pennington *et al*. 2003; Schlosser and Rhaghavendra 2004). AAC has helped children to initiate conversation more frequently, use a wider range of communicative functions, access a broader vocabulary, and increase narrative performance.

### Literacy

Written language problems of children with CP, in both reading and spelling, have become the focus of research over the last decade and studies suggest that literacy difficulties are associated with nonverbal cognition, speech and working memory impairments (Peeters *et al*. 2009, Smith *et al*. 2009, Larsson and Sandberg 2008, Dahlgren-Sandberg 2006). Reading interventions for children with severe speech impairment who use AAC focus on adapting the literacy environment and increasing opportunities for children to participate in literacy activities (Koppenhaver *et al*. 2007, Sturm and Clendon 2004).

### Aims

In summary, children and young people with CP may experience a range of impairments in oromotor control, cognition, language and sensation, which can impact development of communication performance. SLT interventions have been developed to address speech and language impairments and communication limitations, with the goal of facilitating participation in family, educational and community life. Some are beginning to show promising results; however, there is currently no evidence on comparative effectiveness of interventions or to suggest how the needs of young people with CP should be prioritized within rationed SLT provision such as the UK National Health Service (NHS). We aimed to explore how UK SLTs are managing the communication needs of young people with CP. Specifically, we aimed to investigate the following:

- Which areas of need are regularly assessed by UK SLTs?
- How are needs assessed?
- Which areas of need are most frequently addressed in SLT management?
- How do SLTs treat the speech impairments and communication limitations experienced by children and young people with CP?
- Do assessment and management practices vary according to whether therapists also provide dysphagia services, the sector in which they are employed or number of children with CP on their caseload?

## Method

We used RCSLT Guidelines (Taylor-Goh 2005), Communicating Quality 3 (RCSLT 2006) and systemic reviews of intervention evidence (Pennington *et al*. 2003, 2009a) to develop an online survey of the needs of children with CP and a communication difficulty that are commonly addressed by UK SLTs. We asked therapists to provide demographic information on their employment and to select the body functions, communication activities and areas of participation and quality of life they assessed. We listed well known tests for each domain (e.g. receptive language) and asked SLTs to indicate each test they used. We also asked therapists to report any other tests they used, which were not included in the lists, in text boxes. We asked therapists to list the areas of need for which they provided intervention and to prioritize needs for intervention. As the hallmark of CP is a non-progressive motor disorder, we specifically asked therapists about the types of interventions they employed to address children's motor speech disorders and communication difficulties arising from motor impairments. As language interventions are generic, we reduced responder burden by not asking responders to specify language interventions they used. Three SLTs working with children with CP piloted the questionnaire and we made some minor adjustments to the wording of questions following their feedback. The survey was hosted online via Survey Monkey, 5 December 2011–30 January 2012. The survey questions are given in the appendix.

The study was classed as NHS service evaluation, thus no NHS ethical approval was required. Ethical approval was granted by Newcastle University Ethics Committee. We sought approval to circulate the survey around paediatric SLT departments from each UK NHS trust and health board. We contacted the Department of Health (DoH) in England, Welsh and Northern Irish assemblies and the Scottish Parliament to locate all NHS paediatric speech and language therapy departments in the UK. None of these bodies held a central list of children's SLT departments, nor did their constituent strategic health authorities (disbanded in NHS reorganization) and subordinate NHS trusts and health boards. We sent each NHS trust/health board an initial e-mail/web contact form (where online contact details were available on their website) asking if they had a paediatric speech and language therapy department and requested permission to conduct the survey from those that replied positively. We also placed an advert in the RCSLT *Bulletin*. The RCSLT posted a link to the survey on their Facebook and Twitter accounts at four time points whilst the survey was open. Eleven Special Interest Groups (SIGs) advertised the survey to their members. The Association Speech and Language Therapists in Independent Practice, SCOPE (a UK charity that supports and campaigns for disabled people and their families in the UK) and The Communication Trust also advertised the weblink for the survey on its websites/social networks.

### Analysis

We examined the spread of the data using descriptive statistics. In order to examine the association between number of children with CP on therapists’ caseloads we created three groups (numbers of children with CP on caseload = 0–5; 6–20 and > 20). We used two-tailed chi square and Fisher's Exact test to compare groups of SLTs (full time/part time; communication only/communication and dysphagia; number of children with CP on caseload). Statistical significance was set at *p* < 0.05. We performed analyses using SPSS v.19 (SPSS 2011).

## Results

We received 292 responses to the survey. We discarded entries in which the respondent was not an SLT or completed fewer than five questions. The final sample comprised 265 UK SLTs. Not all respondents answered all questions on the survey and we have given the number of respondents answering each question in the results below.

### Demographics

**Figure 1:**
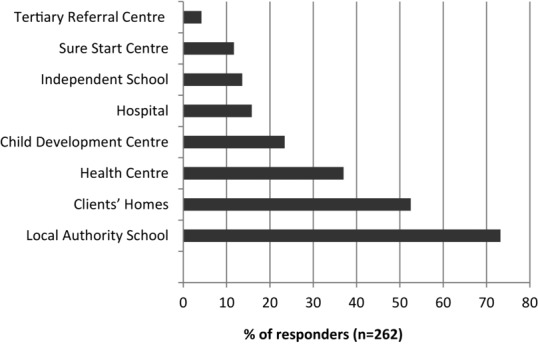
Settings in which SLTs work. Percentages sum to greater than 100 as therapists may work in more than one setting.

**Figure 2:**
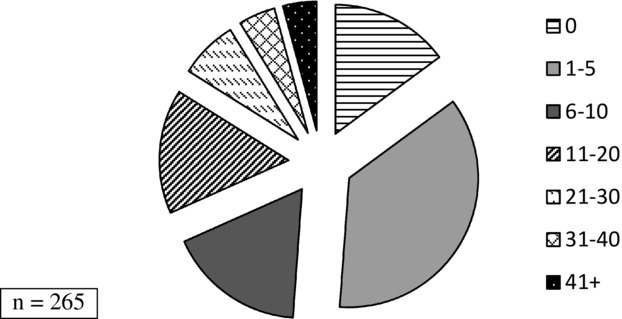
Numbers of children with CP on SLTs’ caseloads including active and review cases.

Just over half of the therapists who responded to the survey worked full time (*n* = 144, 54%). The majority of responders were employed by the NHS (*n* = 228, 86.4%), with small numbers employed by a local authority (*n* = 6, 2.3%), independent school or college (*n* = 13, 4.9%), voluntary sector (*n* = 5, 1.9%) or private practice (*n* = 12, 4.5%). Participants were asked to select all of the sites in which they worked; the most frequent was local authority school with 73.2% (*n* = 194), followed by clients’ homes with (*n* = 139, 52.5%) (figure 1). Approximately half of the respondents worked on communication only (*n* = 136, 51.3%) and half worked on communication and dysphagia (*n* = 129, 48.7%). Overall, just over one third of therapists (*n* = 95) had five or fewer children with CP on their caseload (active and review); 4% (*n* = 11) reported having more than 41 (figure 2). Therapists working on communication only had fewer children with CP on their caseloads than therapists who worked on communication and dysphagia (*Χ^2^* (6, 262) = 67.8; *p* < 0.001).

There was considerable overlap between SLTs working with pre-school children (*n* = 207, 78.1%) and school age children (*n* = 245, 86%). Only 20 (7.5%) respondents reported working with young adults (aged 20–25 years).

The percentages of responders from each UK country were broadly in line with population (Office for National Statistics 2011) and registered SLTs (Health and Care Professionals Council 2012): 199 (74.7%) responders were employed in England (England employs 81.0% of UK registered SLTs), 13 (4.9%) were employed in Wales (4.7% UK registered SLTs), 36 (13.6%) were employed in Scotland (9.5% UK registered SLTs) and 17 (6.4%) of responders were employed in Northern Ireland (4.8% UK registered SLTs). It should be noted, however, that employment data in the UK countries are available for all registered SLTs and not paediatric SLTs separately.

### Assessment practices

**Figure 3:**
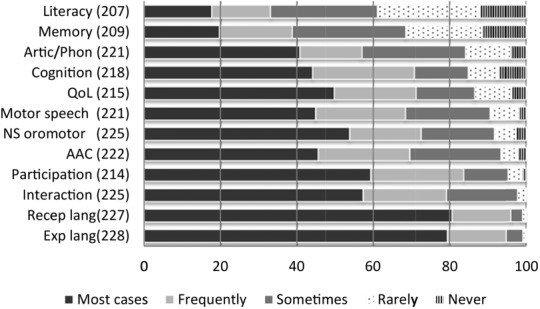
Frequency with which individual domains were assessed at referral/initial assessment expressed as percentages. *n* for each domain is reported in parentheses.

Therapists identified the speech, language and communication domains they assessed when children are first referred, diagnoses are made and areas of need are defined (figure 3). Therapists commented that assessment was individualized and dependent on the child's needs For example, many of the therapists who stated that they did not assess articulation/phonology explained in the free text boxes that this was because the children they worked with were preverbal.

Therapists selected the standardized assessments and published protocols they used and listed other published measures that they used in a free text box. Table1 shows the speech, language and communication assessments used by 10 or more respondent SLTs and the number of other named protocols used by fewer than 10 therapists. Some SLTs commented that children on their caseloads were too young to complete formal assessments or had significant motor or sensory impairments which prevented them from responding in the manner stipulated by the test. The latter led to the SLTs modifying the tests, e.g. enlarging pictures, cutting up response sheets to allow children to point to the target.

**Table 1:** Most frequently used tests employed to assess individual speech, language and communication domains shown as percentage of SLTs responding to each domain

Motor speech skills	Articulation or	Expressive language	Receptive language	Receptive language	Communication
or intelligibility	phonology		(syntax)	(vocabulary and	and interaction
				semantics)	
Observation	Observation	Observation	Observation	Own assessment schedule	Observation
182/210, 86.7%	191/203, 94.1%	192/205, 93.7%	222/224, 99.1%	120/167, 71.9%	203/207, 98.1%
Own assessment schedule	South Tyneside Assessment of Phonology (STAP)^5^	Action Picture Test (RAPT)^9^	Derbyshire Language Scheme (DLS)^18^	British Picture Vocabulary Scales^20^	Own assessment schedule
82/168, 48.8%	157/ 209, 75.1%	170/209, 81.3%	175/210, 86.3%	143/206, 69.4%	109/163, 66.9%
Frenchay Dysarthria Assessment^1^	Own assessment schedule	Clinical Evaluation of Language Fundamentals (CELF)^10^	Own assessment schedule	Bracken Basic Concept Scale^21^	Preverbal Communication Scales (PVCS)^22^
25/177, 14.1%	102/154, 66.2%	134/199, 67.3%	145/177, 81.9%	51/175, 29.1%	119/194, 61.3%
Published voice assessment protocols (e.g. VAP^2^)	Evaluation of Articulation and Phonology (DEAP)^6^	South Tyneside Assessment of Syntactic Structures (STASS)^11^	Evaluation of Language Fundamentals (CELF)^10^	Boehm Test of Basic Concepts^22^	Children's Communication Checklist^23^
14/170, 8.2%	61/177, 34.5%	127/198, 64.1%	130/197, 66.0%	49/174, 28.2%	85/178, 47.8%
Robertson^3^	CLEAR Phonology Screening Assessment^7^	Own assessment schedule	Test of Reception of Grammar (TROG)^19^	Observation	Other published protocol
11/170, 6.5%	34/221, 15.3%	92/153, 60.1%	123/191, 64.4%	24/164, 14.6%	22/104, 21.2%
Nuffield Dyspraxia Programme Assessment Tool^4^	Goldman–Fristoe Test of Articulation^8^'	Renfrew Bus Story^12^	Reynell Developmental Language Scales^15^	Other published protocol	Number of other published assessments = 4 (including one standardized test)
12/221, 5%	18/161, 11.2%	88/181, 48.6%	90/181, 49.7%	11/102, 10.8%	
Other published protocol	Other published protocol	Preschool Language Scales^13^	PreSchool Language Scales (PLS3/4)^13^	Number of other published assessments = 8 (including three standardized tests)	
23/108, 21.3%	44/114, 38.6%	80/172, 46.5%	83/169, 49.1%		
Number of other published assessments = 5 (including one standardized test)	Number of other published assessments = 8 (including one standardized test)	Assessment of Comprehension and Expression (ACE)^14^	Assessment of Comprehension and Expression (ACE)^14^		
		68/175, 38.9%	62/161, 38.5%		
		Reynell Developmental Language Scales^15^	Other published protocol		
		65/175, 37.1%	40/108, 37%		
		Expressive Vocabulary Test^16^	Number of other published assessments = 16 (including seven standardized tests)		
		36/157, 22.9%			
		Expression Reception and Recall of Narrative Instrument (ERRNI)^17^			
		12/152, 7.9%			
		Other published protocol			
		13/100, 13.0%			
		Number of other published assessments = 7 (including three standardized tests)			

Note

1, Enderby and Palmer (2007); 2, Pindzola (1987); 3, Robertson (1987); 4, Nuffield Hearing and Speech Centre/Miracle Factory (2004); 5 = Armstrong and Ainley (2012a); 6, Dodd *et al*. (2006); 7, Keeling and Keeling (2006); 8, Goldman and Fristoe (2000); 9, Renfrew and Hancox (1997); 10, Seme *et al*. (2004); 11 = Armstrong and Ainley (2012b); 12, Renfrew (2010); 13, Zimmerman *et al*. (2011); 14, Adams *et al*. (2001); 15, Edwards *et al*. (2011); 16, Williams (2007); 17, Bishop (2004); 18, Knowles and Masidlover (1982); 19 = Bishop (2003a); 20, Dunn *et al*. (2009); 21, Bracken (2006); 22, Kiernan and Reid (1987); and 23 = Bishop (2003b).

**Table 2:** Interventions provided

	Do you ever provide intervention for …?	
	*n*	Yes (%)
Dysphagia	194	135 (69.6)
Alternative and Augmentative Communication (AAC)	206	194 (94.2)
Interaction/conversation	201	187 (93.0)
Receptive language	209	205 (98.1)
Expressive language	210	203 (96.7)
Speech	207	181 (87.4)
Other	176	16 (18.0)
General oromotor control	199	139 (69.8)
Literacy (excludes SLTs who only work with pre-school)	146	41 (28.1)

Therapists who did not assess cognition, memory or literacy commented that these skills were routinely assessed by other members of the team, such as psychologists and teachers. Those who did assess these areas reported that they did so through observation or using schedules they had developed themselves. These methods of assessment were also used to evaluate quality of life and participation Frequency of observational assessment: AAC competence/proficiency = 72% (*n* = 190); literacy = 87% (*n* = 162); quality of life = 76% (*n* = 204); participatio*n* = 76% (*n* = 204). Frequency of assessment using self-developed protocol: AAC proficiency = 61% (*n* = 178); quality of life and participatio*n* = 22% (*n* = 204); participatio*n* = 22% (*n* = 204). Only six therapists reported using standardized assessments of literacy or phonological awareness and two therapists reported using published measures of quality of life or social participation. Most therapists reported that they accessed teachers’ assessment results to evaluate cognitive development (81%, *n* = 101) and half said that they used teachers’ assessments of children's literacy (50%, *n* = 200).

### Intervention practices

Therapists indicated all domains in which they provided intervention: 69.6% of all respondents reported providing intervention to address dysphagia; 94.2% provided AAC intervention; 93.0% addressed interaction/conversation; 98.1% addressed receptive language; 96.7% provided therapy for expressive language development; 87.4% provided intervention for speech; 69.8% provided therapy for general oromotor control (non-speech); and 28.1% provided intervention for literacy difficulties. In a free text box therapists stated that that they also provided intervention to address attention and listening, saliva control and parental anxiety.

**Figure 4:**
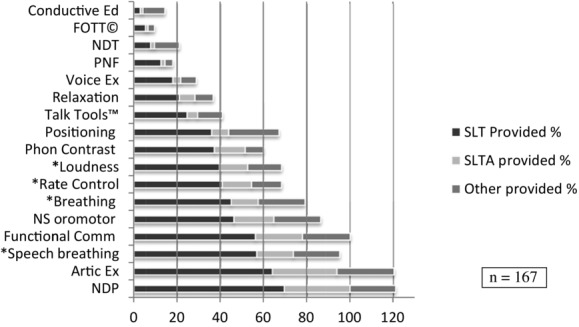
Provision of interventions to improve speech of children with motor speech disorders. Numbers add up to more than 100% as more than one option could be selected for each intervention.

We specifically asked about the interventions provided to reduce speech impairment/improve speech intelligibility of children with motor speech disorders; 207 therapist reported intervening with this aim and 167 (80.7%) reported the interventions they used. The interventions used by more than three responders are shown in figure 4. Three or fewer responders stated that they used PROMPT, EPG or Lee Silverman Voice Treatment® to improve speech; two reported that they taught children a core vocabulary and one reported using Lycra® suits.

**Figure 5:**
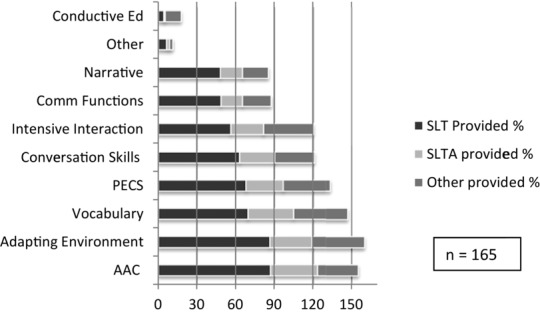
Types of multimodal functional communication intervention provided by SLTs, SLT assistants and others, and their frequency of use. Numbers add up to more than 100% as more than one option could be selected for each intervention.

To assess the broad types of interventions provided to promote communication we asked ‘When working with children with CP do you ever provide intervention that aims to increase children's multimodal, functional communication?’ One hundred and ninety-five responded that they did provide intervention with this aim and 165 (84.6%) of these therapists selected the types of interventions they used (figure 5). Other interventions specified in the free text box that accompanied this question comprised: joint attention, intentional communication; parent training, including Hanen style approaches (Pepper and Weitzman 2004); training speech and language therapy assistant (SLTA) and carers/nursery/school to implement into the environment; social stories; Colourful Semantics (Bryant 1997); teaching grammar, sentence building; and working with education in partnership.

### Association between therapist characteristics and assessment and intervention practice

Most therapists who responded to the survey were employed in the NHS and worked in local authority schools which prevented us from examining the association between place of work and assessment and intervention practice. We found no statistically significant differences between therapists working with communication only and those working with communication and dysphagia in the domains assessed and the use of individual tests. However, the number of children with CP on therapists’ caseloads was associated with assessment and intervention practice. Therapists who had five or fewer children with CP on their caseloads reported assessing articulation and phonology more frequently than therapists seeing higher numbers of children with CP (*Χ^2^* (8, 220) = 35.21; *p* < 0.001). Therapists with five or fewer children with CP on their caseloads were also less likely to assess AAC skills (*Χ^2^* (8, 221) = 29.79; *p* < 0.001) or to provide intervention for AAC (*Χ^2^* (2, 205) = 7.70; *p* = 0.021) or dysphagia (*Χ^2^* (2, 193) = 24.3; *p* < 0.001).

### Speech and language therapy hours

We asked about the breakdown of hours of SLTs and SLTAs when working with children with CP to address communication needs: ‘On average how many speech and language therapy hours per year would a young person with CP receive from your services to address their speech, language and/or communication needs.’ Only about one-third of therapists were able to respond to this question. They reported that preschool children received 0–150 h of therapy from a qualified therapist per year (*n* = 84; median = 20; IQR = 10–30) and school aged children received 1–200 h per annum (*n* = 80; median = 20; IQR = 9.25–30). There were no significant differences in estimated amount of therapy provided by therapists who worked on communication or both communication and dysphagia. A number of respondents reported that input was dependent on the needs of the individual child/family. Given the low response rate to this question and heterogeneity of children with CP the data here must be treated with caution. Even greater uncertainty was expressed about speech and language therapy assistant hours’ input, with fewer than thirty respondents able to give an estimate SLTA time. Given the low level of confidence expressed, data are not reported here.

## Discussion

This study used an online survey to explore how UK SLTs assess and manage the needs of children with CP. We achieved a high response rate from therapists across the four UK countries who worked with children and young people with CP up to the age of 25 years and who were evenly split between those who addressed communication only and those who worked on communication and dysphagia. Respondents working in both communication and dysphagia had more children with CP on their caseloads. Our demographic data suggest that we captured the views of generalist and more specialist therapists across the UK. However, as the survey was completed anonymously, without reference to the responder's employer and employment band, we cannot be certain that respondents are representative of the body of SLTs providing services to children and young people with CP across the UK.

The high response rate to the survey was achieved despite initial recruitment difficulties, which arose because neither the NHS nor governmental departments of health in any of the UK countries hold central records of the trusts/health boards that provide paediatric SLT. A mechanism to locate and contact SLT departments within the NHS would facilitate more representative research within the UK SLT profession. The difficulties in locating the relevant population led us to use social media to advertise the survey and adverts were followed quickly by increases in survey returns. We would recommend engaging with professional bodies and advertising research via social media to facilitate recruitment in this type of survey and other research.

### Assessment

Therapists reported that they commonly assessed each of the domains specified in clinical guidelines (Taylor-Goh 2005), suggesting that children with CP receive comprehensive assessment of their speech, language and communication when they are referred to UK SLT services. It appears that assessments also comply with the NHS goal of personalized medicine, whereby necessary and sufficient tools are used to diagnose the causes of ill-health in children of differing developmental levels (Taylor-Goh 2005, RCSLT 2006), as therapists reported individualizing assessment batteries. A notable example was assessment of articulation/phonology which, therapists explained, was inappropriate for very young children or those with severe or profound intellectual impairment. Therapists also reported using information gathered by other agencies, such as education, when making their initial assessments, especially in the domains of cognition, literacy and quality life. The use of information gathered by others prevents children from undergoing unnecessary assessments and their focus on assessment of oromotor function, speech, language and communication speaks to the unique value of SLT (RCSLT 2006).

Therapists reported using a wide range of range of standardized tests to evaluate impairments in speech and language function. Such tests will allow comparison of the skills of a child with CP against those of typically developing children of the same age. However, as the children involved in test standardization are selected from a population of children without identified developmental issues, these tests do not enable us to compare children with CP to the wider population. Neither do they allow us to attribute reduced scores to particular impairments associated with CP. Some therapists reported that they adapted standardized tests to accommodate children's difficulties in pointing to pictures and verbalizing their responses. As mentioned earlier, changing presentation mode will invalidate test standardization. Nevertheless, adapted tests may still provide an accurate reflection of children's skills in comparison with their typically developing peers if responses are cognitively and motorically simple. For example, expanding quadrants of pictures to enable children to eye or fist point to them may involve a similar type of response as the original test and has been shown to create equivalent scores to the original tests. Other adaptations, however, may alter the cognitive load involved in producing a response and may be testing skills other than the assessment intended (Warschausky *et al*. 2012). More extensive test adaptations should therefore be viewed with caution.

One area of function in which standardized tests require little adaptation but were rarely used was motor speech. Only the Frenchay Dysarthria Assessment (Enderby and Palmer 2007) was used by more than 10 respondents. This assessment has been normed on young people from 12 years of age; the Verbal Motor Production Assessment for Children (Hayden and Square 1999) has norms on children up to 12 years and may provide complementary information on speech motor skills for younger children. A specific omission within the assessment of motor speech was any evaluation of intelligibility. Although the Frenchay includes a section on intelligibility it was used by only 25 respondents and other methods of intelligibility assessment were not specified in free text responses. Therapists may, of course, have been estimating speech intelligibility in their own assessment protocols, but percentage intelligibility estimation has been shown to be unreliable (Hustad 2006). The lack of intelligibility testing has also been noted in other surveys (Miller *et al*. 2011, King *et al*. 2012); nonetheless, its omission here is puzzling given that intelligibility was cited as an area for intervention by most respondents. Clearly, more work is required to raise the profile of (diagnostic) speech intelligibility testing (Miller 2013) and to promote the implementation of intelligibility assessments in clinical practice for diagnostic and outcome measurement purposes.

Standardized tests were rarely used to assess communication activity (such as the ability to ask questions or respond to other people's comments) or communicative participation (use of communication in real world situations; Eadie *et al*. 2006). Early communication was frequently assessed using the Preverbal Communication Scales (PVCS) (Kiernan and Reid 1987), which is not standardized and is no longer in print. The Communication and Symbolic Behavior Scales Developmental Profile™ (CSBS; Wetherby and Prizant 2002) would seem a natural successor to the PVCS as it has the advantage of norms for younger children and has been used successfully in research with children with CP (Coleman *et al*. 2013). But, our results suggest that it has not yet been widely adopted in clinical practice in UK. The Children's Communication Checklist—2 (Bishop 2003b) was used frequently with school aged children and this will identify pragmatic difficulties, especially for children without severe speech disorder. To gain an understanding of the ease with which children communicate in their everyday environments at home, school and in the community irrespective of communication mode (their communicative participation) an additional tool is needed. The Focus on Communication Outcomes Under Six (FOCUS) (Thomas-Stonell *et al*. 2012) is a new assessment of communicative participation that has demonstrated sensitivity to change (Thomas-Stonell *et al*. 2010) and is undergoing further validation (Thomas-Stonell, personal communication, March 2014). However, we know of no current standardized or validated measures of communicative participation for older children. As the goal of SLT intervention for children is independent communication in daily life, this is a serious deficiency for our profession and warrants urgent attention.

In addition to increasing the ease with which children communicate in family, social, educational and community activities, as SLTs we may also aim to help children and young people take part in a broader range of social activities and to do so more frequently. Evaluating the impact of SLT in this way requires measures of global participation, rather than communicative participation. Well validated measures of children's participation now exist (King *et al*. 2004, Jessen *et al*. 2003, Coster *et al*. 2011) but were used by only two respondents in the survey. It is possible that such measures are being used by other members of multidisciplinary teams and their use has not been captured by this survey. But, if this is not the case these measures could be added to our assessment battery to measure valuable, real-world outcomes of SLT interventions.

Many therapists in the survey reported using unstandardized published procedures, such as the STAP (Armstrong and Ainley 2012a), STASS (Armstrong and Ainley 2012b), CLEAR (Keeling and Keeling 2006). Such protocols facilitate consistency of measurement, enabling comparison of children's behaviours across time, but they do not allow us to compare children's development against the norm to show the severity of impairments. Many therapists also reported using protocols that had been developed in-house. These assessments lack validation and may not assess the concepts they are intended to measure. Their localized implementation also prevents comparison of children across services and the generation of knowledge about populations served by speech and language therapists. In all domains covered by the survey therapists most frequently reported assessing children's skills through observation. Children's natural interactions can show how children usually communicate—their communication performance—and can demonstrate the impact of children's environment on their communication, including the influence of communication partners. This is especially important for children with limited intelligibility, who often have restricted patterns of interaction (Dahlgren-Sandberg and Liliedahl 2008, Clarke and Wilkinson 2007, Pennington *et al*. 2009b). However, observation of naturally occurring communication cannot test communication capacity, that is, what children *can* do when given the opportunity. Unless interaction with a range of the child's usual communication partners is observed, it is possible that children's communication performance may be underestimated. Furthermore, natural conversation cannot be replicated to enable comparison across time or people.

Dynamic assessment allows us to evaluate children's capacity when provided with adult assistance and has been used to assess early communicative capacity of children with developmental disabilities (Olswang *et al*. 2013, Letto *et al*. 1994) and language acquisition (Camilleri and Botting 2013). Dynamic assessment techniques may help us bridge the gap between standardized tests, which may be inaccessible to children with motor and sensory impairments, and assessment through observation. Dynamic assessment individualizes assessment and can provide detailed information to guide intervention planning. However, such techniques require meticulous recording and the individualization of assessments prevents comparison across children.

Clinical guidelines recommend the assessment of speech, language and communication function and communicative participation (RCSLT 2005) but not how to undertake this. Our results suggest that many therapists are using a range of tools, but some are relying solely on observation which is inadequate for accurate assessment. Consensus and/or guidance on assessment methods for use with preschool and school aged children could support practice development, by ensuring that across services assessment is comprehensive and that results obtained are valid and reliable. Adoption of such guidance could create a minimum clinical dataset, which would not only have the potential to drive up quality of care for individuals (Svensson-Ranallo *et al*. 2011) but also inform service planning at a local, regional and national level as comparisons across time and place could be made. National data sets could also inform research, by showing the most prevalent communication needs for which we lack evidence of intervention effect.

### Intervention

There was broad agreement between therapists who responded to the survey that dysphagia should be prioritized in speech and language therapy management of children with CP, and that the next two most urgent areas for intervention are interaction skills and receptive language. This prioritization is in line with clinical guidelines (RCSLT 2005, 2006), which state that speech and language therapists’ value is in their promotion of effective communication and language systems and the development of eating and drinking skills, ensuring that children receive adequate nutrition.

Most therapists complied with clinical guidelines (Taylor-Goh 2005, RCSLT 2006) and provided individualized intervention that focussed on communication activity and potentially communicative participation, including teaching conversation skills, vocabulary, narrative and adapting the environment. The types and foci of interventions are wide ranging, which is unsurprising given the heterogeneous nature of CP. Augmentative and alternative communication (AAC) systems were frequently introduced, especially by the potentially more specialized therapists, who had greater numbers of children with CP on their caseloads and who addressed both communication and dysphagia. Intervention also included training for communication partners. Intensive interaction (Hewett and Nind 1993) was used often, and this is to be expected given that around 20% of young people with CP have profound intellectual impairment (Blair *et al*. 2001). However, the use of PECS by over 60% of respondents is surprising, given that PECS was developed for children with autism and the prevalence of autism in CP is estimated to be in the region of only 7% (Christensen *et al*. 2014). Other types of AAC, such as symbol charts or books, may allow children to use a wider range of communicative functions and allow greater independence in communication. The use of PECS for children with CP should be investigated by clinical services to ensure that children are given access to systems that grow with them and enable them to develop a full range of communication skills.

Many of the therapists responding to the survey reported that they provided intervention to improve the speech intelligibility of children with dysarthria. Some of the interventions provided (e.g. breath control, speech breathing, rate control) have been found to be effective in small group studies (Pennington *et al*. 2010, 2013, Fox and Boliek 2012). Many therapists also reported using articulation therapy and non-speech interventions specifically to improve the speech intelligibility in dysarthria. To our knowledge such interventions lack supporting evidence and Communicating Quality 3 (RCSLT 2006) states that SLTs should provide information and advice on why articulation therapy may be inappropriate and counterproductive (p. 280). Several papers caution against the use of non-speech interventions (Maas *et al*. 2008, Powell 2008a, 2008b, Ruscello 2008, Lass and Pannbacker 2008, Wilson *et al*. 2008, Hodge *et al*. 2005). These papers were published in North American journals, which may not be widely available in NHS. However, the issue of non-speech treatments was raised in a recent Cochrane review, which is freely available to the NHS employees (Pennington *et al*. 2009a). The continued implementation of interventions which lack evidence and theoretical underpinning suggests the need for increased efforts to implement evidence-based intervention and wider, perhaps more strategic, dissemination of research at national, regional and local levels.

Wider adoption of evidence based practice may not only serve to reduce the variation in types of interventions offered, but also the variation in the amount of therapy provided by different services. Although many respondents found it difficult to quantify the number of hours spent working with children with CP many did attempt to do so, and estimated input varied by up to 199 h per annum. Variation may arise from heterogeneity of populations served and individualization of provision, but may also reflect real differences in clinical practice across services (Bercow 2008, Gascoigne 2012). Increased awareness of current research, adoption of common assessment protocols and implementation of evidence based practice should help us to map identified needs to evidence-based interventions, and increase equity across NHS services. Such an approach would also show the gaps in evidence, which can be communicated to government departments and funders of research in order to support the generation and testing of new interventions.

### Limitations

This study achieved a high response rate, but voluntary and anonymous response means that we cannot guarantee results are representative of speech and language therapy services across the UK NHS. Selection bias is possible; therapists who seek to drive change may have been more inclined to participate than those who are happy with the status quo. Validity may also be threatened in that the survey asked therapists to report their usual practice but did not capture what therapists actually do. The survey was also limited in its scope. It focussed on initial assessment and subsequent management, but did not ask about outcome measures and how therapists evaluate the success of their interventions. Furthermore, the survey sought only the views of therapists, the voices of families and the young people themselves have yet to be heard.

## Conclusions

The results of the survey suggest wide variation in the assessment and management of the communication needs of children and young people with CP by UK speech and language therapists. Current methods of assessment lack rigour and there is an urgent need to develop consensus in assessment practice across the UK for this client group. Standardization in assessment would allow SLTs in UK to develop a national dataset, which could be used to inform UK health and education policy and drive research. The variation in practice and continued provision of interventions which lack evidence and theoretical underpinning suggest the need for concerted effort to implement evidence based practice. Driving forward change will require action at a national, regional, local and individual level.

## Appendix

UK Survey of Speech and Language Therapy Services for Children with Cerebral Palsy

**Table:**

**ABOUT YOU AND YOUR JOB**				
**1. Please state the age range of clients you work with (tick all that apply):**				
Preschool	School age (5-19 years)	Young adults (20-25)		
**2. Do you work:**				
Full time	Part time			
**3. In which setting are you *based***				
Health centre	District Hospital	Child Development Centre		
Tertiary referral centre	LA School	Independent school		
**4. In which settings do you work (tick all that apply)**				
Health centre	District Hospital	Child Development Centre		
Clients’ homes	Sure Start centre	Tertiary referral centre		
LA School	Independent school			
**5. Please tell us in which sector you are employed:**				
NHS	LA	Higher/further education		
Independent school/college	Voluntary sector	Private practise		
**6. How many children with CP do you currently have on your caseload (please include both active and review cases)?**				
0	1-5	6-10	11-20	
21-30	31-40	41+		
**ASSESSMENT**				
**7. Children with CP may have a range of speech, language and communication impairments**.				
***Most cases***	***frequently***	***sometimes***	***rarely***	***never***
AAC proficiency/competence	Oro-Motor skills (non-speech)	Motor speech	Articulation/Phonology	Expressive language
Receptive language	Pragmatics/interaction (including pre-intentional communication)	Cognition	Memory	Literacy
**8. Which assessments do you use with children who have CP?**				
***RECEPTIVE LANGUAGE***:				
***Syntax***				
***Most cases***	***frequently***	***sometimes***	***rarely***	***never***
Clinical Evaluation of Language Fundamentals (CELF)	PreSchool Language Scales (PLS3/4)	Derbyshire Language Scheme (DLS)		
Reynell Developmental Language Scales	Assessment of Comprehension and Expression (ACE)	Test of Reception of Grammar		
Other published protocol (please name)	Observation	Own assessment schedule		
***Vocabulary and semantics***				
***Most cases***	***frequently***	***sometimes***	***rarely***	***never***
British Picture Vocabulary Scales	Peabody Picture Vocabulary Test	Boehm Test of Basic Concepts	Bracken Basic Concept Scale	
Test of Word Knowledge (TWOK) Observation	Other published protocol (please name)	Own assessment schedule		
***SPEECH***				
***Please tell us how you assess the speech of children who use spoken language***				
***Articulation/Phonology***				
***Most cases***	***frequently***	***sometimes***	***rarely***	***never***
Diagnostic Evaluation of Articulation and Phonology (DEAP)	Goldman Fristoe Test of Articulation	South Tyneside Assessment of Phonology (STAP)		
Other published protocol (please name)	Observation	Own assessment schedule		
***Motor speech and intelligibility***				
***Most cases***	***frequently***	***sometimes***	***rarely***	***never***
Children's Speech Intelligibility Measure (CSIM)	Verbal Motor Production Assessment for Children (VMPAC)	Frenchay	Robertson	Assessment of Intelligibility of Dysarthric Speech (ASSIDS)
TOCS+	Published voice assessment protocols (e.g. VAP, GRBAS)	Other published protocol (please name)	Observation	Own assessment schedule
***EXPRESSIVE LANGUAGE***				
***Most cases***	***frequently***	***sometimes***	***rarely***	***never***
Clinical Evaluation of Language Fundamentals (CELF)	Preschool Language Scales	Reynell Developmental Language Scales	Assessment of Comprehension and Expression (ACE)	
Expressive Vocabulary Test	Renfrew Bus Story	South Tyneside Assessment of Syntactic Structures (STASS)	Expression Reception and Recall of Narrative Instrument (ERRNI)	
Rapid Action Picture Test (RAPT)	Other published protocol (please name)	Observation	Own assessment schedule	
***COMMUNICATION: INTERACTION***				
***Most cases***	***frequently***	***sometimes***	***rarely***	***never***
Children's Communication Checklist (CCC)	Preverbal Communication Scales	Communication and Symbolic Behavior Scales Developmental Profiles (CSBS DP)	Children's Communication Checklist (CCC)	
Other published protocol (please name)	Observation	Own assessment schedule		
***AAC PROFICIENCY/COMPETENCE***				
***Most cases***	***frequently***	***sometimes***	***rarely***	***never***
Observation	Own assessment schedule	Published assessment/protocol (please name)		
***LITERACY***				
***Most cases***	***frequently***	***sometimes***	***rarely***	***never***
Teacher assessment	Observation	Own assessment schedule	Published assessment/protocol (please name)	
**COGNITION**				
***Most cases***	***frequently***	***sometimes***	***rarely***	***never***
Observation	Own assessment schedule	Published assessment/protocol (please name)		
**INTERVENTION**				
**9. When working with children with CP do you provide INTERVENTION for the following: (type and models of treatment will be discussed below)**				
***Most cases***	***frequently***	***sometimes***	***rarely***	***never***
Oromotor control (nonspeech)	Articulation	Phonology	Motor speech	Receptive language
Expressive language	Pragmatics/interaction	Literacy	AAC use	Other (please describe)
**INTERVENTION FOR CHILDREN WITH CP AND SPEECH DISORDER**				
**10. If you provide intervention that aims to increase general oromotor control which types of treatment do you use?**				
***Most cases***	***frequently***	***sometimes***	***rarely***	***never***
Bobath/Neurodevelopmental therapy	Castillo-Morales/Orofacial Regulation Therapy	Conductive education	Facial Oral Tract Therapy (FOTT)©	
Nonspeech oromotor exercises	PNF	Positioning techniques	PROMPT	
Relaxation	Talk Tools™	Voice exercises		
**11. If you provide intervention that aims to reduce speech impairment/increase speech intelligibility which types of treatment do you use?**				
***Most cases***	***frequently***	***sometimes***	***rarely***	***never***
Articulation exercises	Breathing exercises	Breath support for speech/speech breathing	Bobath/Neurodevelopmental therapy	Castillo-Morales/Orofacial Regulation Therapy
Conductive education	EPG	Facial Oral Tract Therapy (FOTT)©	Functional communication (e.g. acquisition and use of individual functions of communication, conversation skills)	Maintaining loudness
Nonspeech oromotor exercises	PNF	Pacing/rate control	Phonological contrast therapy	Positioning techniques
PROMPT	Relaxation	Talk Tools™	Voice exercises	LeeSilverman Voice Therapy®
**12. If you provide intervention that aims to increase children's multimodal, functional communication which types of treatment do you use:**				
***Most cases***	***frequently***	***sometimes***	***rarely***	***never***
AAC	Adapting the communication environment	Conductive education		
Functional communication (e.g. acquisition and use of individual functions of communication, conversation skills)	Intensive interaction	PECS		
If you provide formal, published training for communication partners please tell us which training programmes you use: (space provided for completion)				
**TEAM WORKING**				
**13. When working with children who have CP do you work with (tick all that apply):**				
Physiotherapists	Occupational therapists	Psychologists	Psychiatrists	
Community paediatricians	Neurodisability paediatricians	Neurologists	ENT surgeons	
Specialist Health Visitors	Specialist Nurses	Specialist service for people with hearing impairments	Specialist service for people with visual impairments	
Early Years Support Worker	Teachers	Teaching assistants	Sure Start	
Voluntary sector	Parents			
**14. Do you have access to expert AAC assessment and advice (tick all that apply):**				
Yes - in department	Yes – secondary referral	Yes – tertiary referral	Yes – referral to ACE Centre	Yes – referral to AAC manufacturers
